# Liquid Biopsies for Ovarian Carcinoma: How Blood Tests May Improve the Clinical Management of a Deadly Disease

**DOI:** 10.3390/cancers11060774

**Published:** 2019-06-04

**Authors:** Roxane Mari, Emilie Mamessier, Eric Lambaudie, Magali Provansal, Daniel Birnbaum, François Bertucci, Renaud Sabatier

**Affiliations:** 1 CRCM-Predictive Oncology Laboratory, Institut Paoli-Calmettes, Inserm, CNRS, Aix-Marseille Univ, 232 Boulevard Sainte Marguerite, 13009 Marseille, France; Roxane.MARI@ap-hm.fr (R.M.); emilie.mamessier@inserm.fr (E.M.); lambaudiee@ipc.unicancer.fr (E.L.); daniel.birnbaum@inserm.fr (D.B.); bertuccif@ipc.unicancer.fr (F.B.); 2 CRCM-Department of Medical Oncology, Institut Paoli-Calmettes, Inserm, CNRS, Aix-Marseille Univ, 232 Boulevard Sainte Marguerite, 13009 Marseille, France; 3 Department of Medical Oncology, Institut Paoli-Calmettes, 232 Boulevard Sainte Marguerite, 13009 Marseille, France; provansalm@ipc.unicancer.fr

**Keywords:** ovarian cancer, liquid biopsies, circulating tumor cells, circulating tumor DNA, circulating miRNA, circulating exosomes

## Abstract

Ovarian cancers (OvC) are frequent, with more than 22,000 new cases each year for 14,000 deaths in the United States. Except for patients with *BRCA1* or *BRCA2* mutations, diagnostic methods, prognostic tools, and therapeutic strategies have not much improved in the last two decades. High throughput tumor molecular analyses have identified important alterations involved in ovarian carcinoma growth and spreading. However, these data have not modified the clinical management of most of patients. Moreover, tumor sample collection requires invasive procedures not adapted to objectives, such as the screening, prediction, or assessment of treatment efficacy, monitoring of residual disease, and early diagnosis of relapse. In recent years, circulating tumor biomarkers (also known as “liquid biopsies”) such as circulating tumor cells, circulating nucleotides (DNA or miRNA), or extracellular vesicles, have been massively explored through various indications, platforms, and goals, but their use has not yet been validated in routine practice. This review describes the methods of analysis and results related to liquid biopsies for ovarian epithelial cancer. The different settings that a patient can go through during her journey with OvC are explored: screening and early diagnosis, prognosis, prediction of response to systemic therapies for advanced stages, and monitoring of residual subclinical disease.

## 1. Introduction

With more than 22,000 new cases each year for 14,000 deaths in the United States, ovarian cancer (OvC) is the fifth cause of death by cancer for female patients [1]. Despite changes in recommendations and treatments over the past two decades, the prognosis has been only slightly changed, with survival rates of less than 30% for advanced stages. Improved knowledge of tumor biology, derived in particular from international initiatives such as The Cancer Genome Atlas and the Ovarian Cancer Association Consortium [2,3,4], has not brought any benefit for most of the patients. Only those with homologous recombination deficiency, notably *BRCA1* or *BRCA2* mutations, have seen dramatic survival improvements in the last years [5,6,7,8]. It is, indeed, difficult to understand the molecular complexity of OvC in an individual patient. Genomic studies published to date have been based on tumor tissue analyses that must be interpreted with caution due to the spatial heterogeneity of these tumors [9]. These analyses are based on tissue samples, which require invasive procedures (surgery, radio-guided biopsies) that can be associated with complications for about 5% of patients, cannot be repeated at several time points [10]. Moreover, CA125 and HE4, the two routinely-used serum markers, have low sensitivity and specificity for early stage diseases, preventing their use for screening and diagnosis [11]. The development of reliable biomarkers identified by non-invasive procedures is therefore an absolute necessity. This process will allow for better assessment and monitoring of patients treated for OvC, as well as the possibility of exploring new ways to understand OvC complexity from early stages to late relapses [12]. In recent years, hope has come from circulating tumor markers (also known as “liquid biopsies”), including circulating tumor DNA (ctDNA), circulating tumor cells (CTC), circulating miRNA, and circulating exosomes [13,14,15,16].

We describe here the interest of liquid biopsies in screening and early diagnosis, prognosis, prediction of treatment response, detection of minimal residual disease, and early diagnosis of relapse for ovarian carcinoma. We have conducted a search for published articles referring to these questions (research date: 28 February 2019). The current literature evaluating liquid biopsies in the context of OvC has been reviewed.

## 2. Methods

The review was done following the PRISMA (Preferred Reporting Items for Systematic reviews and Meta-Analyses) guidelines [17] (see Table S1).

The research was conducted in PubMed (https://www.ncbi.nlm.nih.gov/pubmed), using the following MESH words: “cell-free tumor DNA” or “circulating tumor DNA”, “circulating tumor cells”, “circulating microRNA” or “cell-free microRNA” or “serum miRNA”, “circulating extracellular vesicles” or “circulating exosomes” and “ovarian neoplasm”. All articles in English indexed from 2000 to 28 February 2019 were eligible.

Articles were selected independently by two authors, RM and RS. We first selected papers of interest according to the article’s title. A further selection was then done based on abstract analysis. After complete manuscript reading of the selected articles, we eliminated all papers outside the field of this review, i.e., those not focused on liquid biopsies or ovarian carcinomas. All remaining original articles, and some reviews, were included in this review.

## 3. Results

We screened 1213 articles after the PubMed search, 346 of which were eligible after title filtering, 199 after abstract-based selection, and 146 finally included (Figure S1).

### 3.1. Circulating Cell-Free DNA and Circulating Tumor DNA

The presence of cell-free DNA (cfDNA) circulating in biological fluids is a physiological phenomenon, secondary to DNA release, mainly by apoptotic or necrotic cells, but also by direct secretion and exocytosis [18]. In the case of cancer, part of the circulating cfDNA corresponds to the DNA released by cancer cells [19]. This ctDNA carries tumor specific molecular alterations, such as mutations [20], translocations, loss of heterozygosity [21], copy number alterations [22], and methylations [23]. Isolation of ctDNA is feasible for several tumor localizations, including OvC [24,25]. Moreover, it can be improved after fragment size selection [26]. One of the main advantages of ctDNA is that it is supposed to better reflect tumor heterogeneity than tissue biopsies [27]. Concerning OvC, cfDNA, and ctDNA analyses have been widely described during the last decade. Their potential applications range from screening to the prediction of response to systemic therapies and monitoring of subclinical disease (Table 1). Translational and clinical studies are underway to confirm the clinical validity and utility of these biomarkers.

#### 3.1.1. Screening and Early Diagnosis

CA125 has insufficient sensitivity and specificity to be a reliable screening biomarker. To select which patients should undergo surgery requires a biomarker that would be more sensitive than CA125 and HE4 and able to distinguish OvC from benign lesions. A study focused on chromosomal instability showed how it is possible to distinguish malignant from benign tumors using the copy number alteration profiling of cfDNA [28]. Sixty-eight women with an adnexal mass and 44 healthy women were enrolled. Among the 68 adnexal masses, 57 were invasive or borderline carcinomas, and 11 were benign masses. Using whole-genome low-coverage sequencing, it was possible to distinguish benign from malignant tumors with an area under the curve of 0.89. Non-invasive pre-natal testing was also used to detect OvC [22]. Indeed, pre-symptomatic malignancies have been incidentally detected thanks to non-invasive pre-natal testing that was supposed to detect fetal aneuploidy by sequencing cfDNA in the maternal plasma. The test was used to detect OvC in non-pregnant women. Thirty-two plasma samples from women recently diagnosed with OvC and 32 healthy controls were studied. Whole-genome sequencing detected 40% (13/32) of cancer cases. Another study compared the screening value of ctDNA to CA125 and computed tomography scans [29]. Whole-exome sequencing and targeted gene sequencing by quantitative PCR (polymerase chain reaction) were used on tumor DNA to identify tumor specific mutations. Droplet digital PCR was then used on cfDNA, and ctDNA was identified for 93.8% of patients. For six cases, a tumor was only detected by ctDNA, whereas CT scans remained negative, with an average advance of 7 months for ctDNA compared with radiological explorations. Methylation markers could also be of interest for screening purposes, by differentiating benign from malignant tumors. As frequently observed in cancer, methylation of the CpG islands is implicated in the development of OvC, by repressing transcription of tumor suppressor genes. Analysis of methylated DNA has several advantages, including a good reproducibility at various time points because the methylation pattern of a single gene is conserved throughout disease progression. Furthermore, methylated DNA is chemically stable and unaffected by sample collection and shipping conditions. However, DNA methylated patterns are often observed at low abundance and their study requires a high signal-to-noise ratio, which could be, for instance, increased by techniques such as digital PCR [43]. As an example, methylation profiling of two promoters—*PGR*-*PROX* and *RASSF1A*—was used to differentiate benign from malignant tumors, with a sensitivity of 80% and a specificity of 73% [30]. In a recent study including more than 400 patients, a three-DNA-methylation-serum-marker panel could differentiate high grade serous OvC patients from other patients with a high specificity (90.7%), but insufficient sensitivity (41.4%). Accuracy was, nevertheless, improved when analysis focused on CA125-negative cases (87.5% specificity and 63.6% sensitivity) [44]. Moreover, because most genomic alterations associated with OvC (*TP53* mutations, copy number alterations, DNA repair deficiency) can be identified in precursor lesions called STICs (serous tubal epithelial carcinomas), monitoring of these alterations for patients at a high risk of OvC (such as *BRCA1/2* mutation carriers) may improve early diagnosis for these patients and may allow prevention by adnexal removal [45].

#### 3.1.2. Prognostic Value

Qualitative and quantitative analyses of circulating genomic alterations can refine OvC prognosis. In a series of 62 OvC patients, pre-operative cfDNA concentration was correlated to prognosis (*p* = 0.02 for progression-free survival (PFS) and *p* = 0.01 for overall survival (OS)) [31]. Circulating tumor DNA was also evaluated after surgery and during adjuvant chemotherapy in 22 patients treated for OvC [29]. Undetectable rates of ctDNA six months after completion of treatment were associated with a better PFS (*p* = 0.0011) and OS (*p* = 0.0194). The median PFS was 32 months for ctDNA-negative patients versus 6 months for patients with detectable ctDNA after treatment. No correlation with prognosis was observed for pre-chemo ctDNA levels. Contradictory results were observed in a series of 268 patients including 164 OvC. Survival was correlated to pre-treatment ctDNA for these 164 patients, with a median OS of 3.1 years in the case of high ctDNA levels versus 4 years for patients with low ctDNA levels [32].

Methylation-specific PCR was used to analyze the acquisition of *hMLH1* methylation in ctDNA [33]. *hMLH1* is a DNA mismatch repair gene, and its loss-of-function can lead to a DNA mismatch repair (MMR) deficiency called microsatellite instability. One hundred and thirty-eight samples from the SCOTROC1 phase III clinical trial (evaluating carboplatin plus paclitaxel versus carboplatin plus docetaxel for first-line treatment of epithelial ovarian carcinoma) were analyzed to assess methylation of *hMLH1* CpG islands before chemotherapy and at relapse. A significant increase in *hMLH1* methylation was observed, from 12% before chemotherapy to 33% at relapse. The acquisition of *hMLH1* methylation at relapse could be detected for 25% of relapse samples, was responsible of a loss of MMR-dependent apoptotic response and was correlated to post-progression survival in multivariate analysis (Hazard Ratio (HR) = 1.99; *p* = 0.007).

#### 3.1.3. Prediction and Monitoring of Response to Treatment

Even though most high grade OvCs are usually sensitive to platinum-based chemotherapy in a first-line setting, a quarter are initially resistant, and all relapsing patients sooner or later become resistant. Unfortunately, no biological surrogate marker of platinum-resistance has been prospectively validated and can be used in clinical routine practice. Circulating biomarkers could, therefore, be of interest in this setting.

Analysis of *TP53* mutations in cfDNA with digital PCR was used to monitor tumor burden and to follow the response to treatment in 40 patients (mainly relapse cases) treated for high grade serous OvC [34]. *TP53* mutant allele fraction concentration, contrary to CA125, was associated with tumor volume assessed using CT scans and time-to-progression. A decrease in *TP53* mutant allele fraction concentration superior to 60% was an independent predictor of time-to-progression in multivariate analysis (HR = 0.22, *p* = 0.008). A response to chemotherapy was seen earlier with ctDNA (median time to nadir 37 days) than with CA125 (median time to nadir 84 days). In another study using Sanger sequencing, a *TP53* mutation was found in tumor for 41 of 61 patients recently diagnosed with advanced high grade serous OvC [35]. All identified *TP53* mutations were detected in the corresponding plasma samples. Patients were divided into two groups (low versus high plasma *TP53* mutant allele count level at three months after chemotherapy completion). Time-to-progression was shorter for cases with a high mutant allele count (*p* = 0.038).

Recently, PARP (poly (ADP-ribose) polymerase) inhibitors have modified the standard of care for OvC. These compounds have been approved for post-chemotherapy maintenance for platinum-sensitive relapses whatever the *BRCA1/2* status, and for *BRCA1/2*-mutated cases after first-line treatment [5,6,7,8]. PARP is involved in base excision repair mechanisms, and its inhibition favors non-homologous end-joining repair (NHEJ), frequently associated with errors and cell death. In the case of homologous recombination deficiency, the inhibition of NHEJ leads to synthetic lethality and cell apoptosis. *BRCA1* and *BRCA2* mutations were assessed in plasma samples from 121 patients with OvC by panel-based next generation sequencing (NGS) [36]. Thirty of the 121 patients were found to carry *BRCA1* or *BRCA2* mutations, including seven with exclusively somatic mutations. This may lead to systematic assessment of somatic mutation in ctDNA for patients with no germline mutation and failures or false negatives in tumor genomic assessment. Moreover, ctDNA can be a surrogate marker of PARP inhibitor efficacy. The *TP53* mutant allele fraction, explored by targeted amplicon deep sequencing, in 18 patients treated with the PARP inhibitor rucaparib in the ARIEL2 phase II trial was used to monitor treatment response [37]. None of the five patients with a moderate *TP53* mutant allele fraction decrease (below 50% compared to initial rate) after the first cycle of treatment achieved a RECIST (Response Evaluation Criteria In Solid Tumors) response. By contrast, seven of the nine patients with a high *TP53* mutant allele fraction decrease (>50% compared to initial rate) achieved partial response according to RECIST criteria. Other alterations in the homologous recombination pathway can also be observed in OvC and are correlated to PARP inhibitor efficacy [46,47,48]. Identification in plasma of alterations of other homologous recombination associated genes is currently underway in a multicenter prospective study (CIDOC (*CIrculating tumor DNA as an early marker of recurrence and treatment efficacy in Ovarian Carcinoma*) trial, NCT03302884).

Secondary intragenic reversion mutations of *BRCA1/2* restore protein function and yielded an acquired resistance to PARP inhibitors. Detection of reversion mutations in ctDNA may predict treatment response. Reversion of *BRCA1/2* germline mutations was assessed in ctDNA for 30 patients with high grade serous ovarian carcinoma [38]. Reversion mutations were found in tumor tissues for five patients, all of which had recurrent disease. Reversion mutation was detected in the plasma of three patients; all were resistant to platinum-based chemotherapy or PARP inhibitors. Another study assessed *BRCA* reversion mutation—using NGS of cfDNA—in *BRCA* mutation carriers treated with rucaparib [39]. Cell-free DNA was extracted from plasma samples for 112 patients with germline or somatic *BRCA1/2* mutations, before and after rucaparib treatment. Ninety-seven patients displayed *BRCA1/2* mutations. Among them, 48 were platinum-sensitive, 38 were platinum-resistant, and 11 were platinum-refractory. *BRCA1/2* reversion mutations were found in pre-treatment ctDNA from eight patients: 2/11 (18%) of platinum-refractory cancers, 5/38 (13%) of platinum-resistant cancers, and 1/48 (2%) of platinum-sensitive cases. Patients without *BRCA1/2* reversion mutations had a longer PFS after rucaparib treatment than patients with reversion mutations—a median PFS of 9 months versus 1.8 months (HR = 0.12, *p* < 0.0001). To study the acquired resistance, samples collected at multiple time points between pretreatment and progression and after progression were also sequenced. Reversion mutations were identified in eight additional patients. In four of these eight patients, reversion mutations were detected prior to progression (median time of 3.4 months). The remaining four patients had reversion mutations detected after progression. Similar results were obtained in another set of 24 mutated patients, including 19 with platinum-resistant OvC and five treated for breast cancer [40]. Panel-based NGS identified *BRCA1/2* reversion mutations in four patients with OvC, out of which one was also resistant to PARP inhibitor therapy. This patient displayed nine different *BRCA2* reversion mutations.

Specific molecular alterations can also be used to monitor response to treatment. As an example, *FGFR2* (fibroblast growth factor receptor 2) was monitored using ctDNA in a patient treated for an ovarian cancer and carrying a fusion transcript involving this gene [41]. During four years, ctDNA carrying the *FGFR2* fusion was always detectable by RT-PCR, whereas CA125 was elevated for only 3 of 28 measurements, while the patient had recurrences proven by biopsies. In a small series of ten patients, tumor-specific chromosomal rearrangements were identified in plasma using qPCR [42]. Persistence of such circulating rearrangements after surgery was consistent with residual disease.

Finally, DNA methylation can also be used to predict response to treatments. A three-marker model exploring the methylation of three serum markers had a better predictive value to evaluate the response to platinum-based treatment than CA125 (identification of 78% of responders to chemotherapy and 86% of non-responders with ctDNA versus 20% and 75% for CA125, respectively) [44].

**Figure 1:**
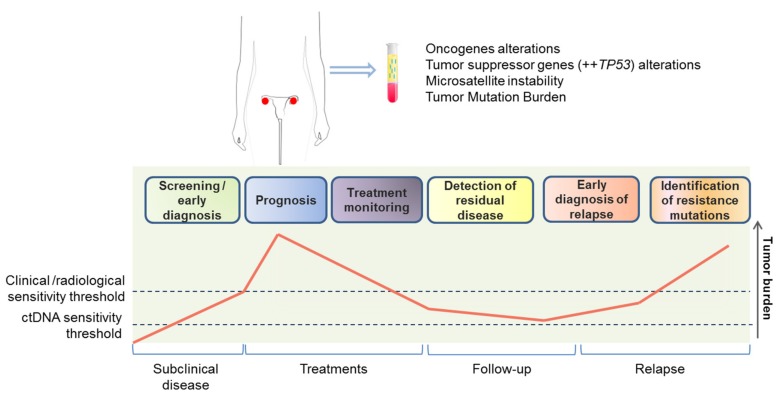
Circulating tumor DNA applications for ovarian cancer management. Detection and monitoring of genomic alterations with approved therapies or phase 2/3 clinical trials available (**top**). Potential use of circulating tumor DNA for various clinical settings (**bottom**); inspired from [49].

The study of ctDNA can thus be a good approach for quantitative analysis and clinical monitoring of OvC (Figure 1). However, because it is mostly released by dying cells, it may be of limited interest for the understanding of OvC biology. Moreover, because it does not allow analyses at other levels (RNA, proteins, metabolites) complementary technologies have been developed.

### 3.2. Circulating Tumor Cells

**Figure 2:**
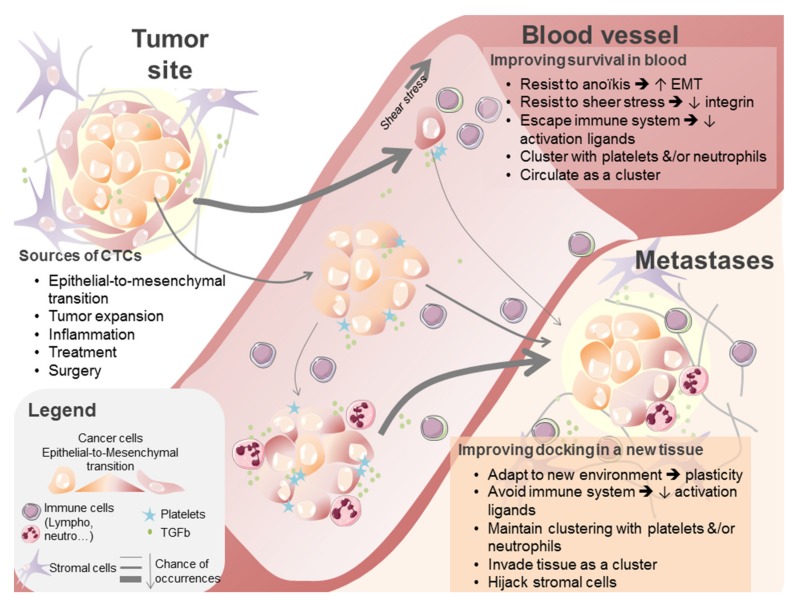
Description of circulating tumor cell (CTC) origins, biological features required to survive blood stream pressures, and abilities facilitating metastases development.

Like DNA, living tumor cells can be released in the bloodstream, from the early onset of cancer development to advanced stage diseases, both by the primary tumor and secondary lesions [13]. Some of these circulating tumor cells (CTCs) may be able to colonize distant sites and may be responsible for loco-regional and distant metastases after going through various degrees of epithelial-to-mesenchymal transition (EMT) [50,51]. Environmental stresses encountered in the bloodstream (flow, immune cells) are indeed not adapted to epithelial cells, and only tumor cells with specific characteristics can survive under these conditions [52]. Recent data suggest that neutrophils are involved in CTC protection in this environment [53,54] as was already shown for platelets, macrophages, and chemokines [13,55]. In all these cases, TGFβ is a key player involved in the induction of tumor cells plasticity, migration, and invasion abilities (Figure 2).

CTCs can be detected after enrichment using various methods based on at least one physical (size, density, electric charges, deformability, invasive capacity) and/or biological features (expression of epithelial markers and negative selection of hematopoietic markers) [56,57,58]. After enrichment, CTCs can be detected by immune–cytological assays (epithelial protein expression), genomic assays (epithelial mRNAs), and functional methods [59]. Concerning OvC, this has been described from the early 2000 s. CTCs have been identified using microbeads coated with the epithelial marker MOC-31 [60] or a combination of cytokeratins and EGFR [61]. Both methods had a detection rate below 20%. Because CTCs can undergo EMT and acquire some capacities such as plasticity, migration and invasion, and resistance to anoikis [62], these epithelial markers may be of limited sensitivity. Most of CTCs are supposed to undergo only a “partial” EMT, and detection can be improved by combining epithelial and mesenchymal markers [63,64]. Nevertheless, CTC collection and analysis cannot be easily implemented in routine practice due to pre-analytics issues. CTC half-life is indeed short (around 4 h) once the blood is drawn [65]. This requires one to process blood samples, with specific equipment, immediately after venipuncture. Novel, exhaustive, easy, convenient, and platform-free technologies are now available (Screencell™, Sarcelles, France). Because these technologies are still recent, there is currently no available meta-analysis using them in the context of OvC.

Despite these limitations, CTC analysis can offer crucial data, both from quantitative and qualitative points of views [13]. Concerning ovarian carcinoma, CTCs have been described as prognostic and their kinetics under treatment as correlated to response to chemotherapy. CTC single cell analyses may also offer new models to explore OvC biology (Table 2).

#### 3.2.1. Quantitative Analyses

Like ctDNA, CTC identification can discriminate healthy individuals and patients with benign adnexal tumors from cases with ovarian carcinoma. In a series of 200 cases with blood samples available, only one of 39 healthy subjects was CTC-positive versus 24.5% of patients with OvC when exploring by RT-qPCR the expression of genes differentially expressed between tumor tissues and leucocytes [66]. Comparing benign tumors with less than 5% of CTC-positive (by flow cytometry) cases versus 88.6% for patients with OvC led to similar observations [67]. In a more recent series of 87 patients with indeterminate adnexal masses, CTCs enrichment and detection (using physical properties of CTCs and CD45/CK/EpCAM immunostainings) was able to discriminate benign tumors from OvC with a 77.4% sensitivity and 100% specificity [68]. Detection rates were similar through OvC pathological subtypes but were correlated to grade [61,67].

It is now well established that CTC detection has a prognostic impact [79,80]. CTC identification is clearly correlated to tumor burden, with higher CTC detection rates for advanced stages of disease versus localized tumors. In studies where CTCs were selected using a cell adhesion matrix-based platform, combining functional cell enrichment and identification with flow cytometry, 50% of stage I tumors were CTC-positive versus 96% for stage IV [67,69]. CTC levels were also higher in the late stage than in early stage patients [70]. CTC detection seemed to be superior to CA125 assessment for detecting early stage tumors [67]. CTC detection is correlated to response to chemotherapy and survival [67]. Changes in CTC counts were highly correlated to response to treatment and CTC increase during follow-up (79.5% of cases) was more sensitive than CA125 (67.6% of cases) to predict progression or relapse [69].

In a series of 129 untreated patients, CTC detection was correlated to OS and PFS both for early stages and advanced tumors [67]. This impact on survival has also been observed in other studies. In a study using an immunomagnetic assay (AdnaTest BreastCancer, QIAGEN, Hilden, Germany) based on EpCAM/MUC1 protein expression and *EpCAM/MUC1/ERBB2* expression by RT-PCR, 19% of the 122 analyzed patients were CTC-positive at diagnosis and 27% after platinum-based chemotherapy [71]. CTC detection at one of these time points was associated with a shorter OS. A study focusing on *ERCC1* expression, coding for a protein involved in DNA repair and resistance to platinum compounds, in CTCs collected before debulking surgery using the AdnaTest Ovarian Cancer (QIAGEN, Germany) [72] observed that 14% of patients were CTC-positive and that *ERCC1+* CTCs were associated with outcome. Of note, this correlation was not observed with *ERCC1* expression on tumor tissue, suggesting that *ERCC1* might be a marker for drug resistant CTCs. Other studies exploring CTC detection by other methods showed similar results both in primary and recurrent settings [66,73,74,75,81]. All these data have been gathered in two meta-analyses, which drew similar conclusions. A total of 1129 patients from 11 studies [82] showed that CTC detection at baseline was associated with both OS (HR = 1.61; 95% CI [1.22–2.13]) and PFS (HR = 1.44; 95%CI [1.18–1.75]). Subgroups analyses based on the methods of detection suggested that correlation to OS was only observed when RT-PCR (with various gene panels) was used (HR = 2.02) and not in studies using CellSearch^®^ (Menarini Silicon Biosystems Inc., Huntington Valley, PA, USA) and other immunocytochemistry-based technologies (HR = 1.15 and 1.09, respectively). A more recent meta-analysis, focused on CTCs and disseminated tumor cells (DTC) in the bone marrow, included 1184 patients from eight studies [83]. CTC-positive patients had a poorer outcome with reduced OS (HR = 2.09; 95%CI [1.13–3.88]) and PFS (HR = 1.72; 95%CI [1.32–2.25]). DTC detection in the bone marrow also showed a correlation to OS and PFS but with lower HRs (1.61 and 1.44, respectively). These results are consistent with that of another meta-analysis focused on the same topic in which HRs were 1.97 (OS) and 2.52 (PFS) for CTCs versus 1.89 (OS) and 1.60 (PFS) for bone marrow-DTCs [84].

Because most patients with complete remission after initial therapies relapse within a few years, assessment of minimal residual disease (MRD) can be of interest to select patients needing maintenance treatments. CTCs may be good surrogates of MRD. Analysis of the *ERCC1* expression of paired samples before and after chemotherapy enhanced the overall CTC-detection rate up to 17%. The presence of *ERCC1* + CTCs after chemotherapy correlated with platinum-resistance (*p* = 0.01), reduced PFS (*p* = 0.0293), and OS (*p* = 0.0008) [76]. Interestingly, modifications of *ERCC1* expression by CTCs were also prognostic, with the worst outcome found for cases that remained *ERCC1*+ after chemotherapy. A study collected blood samples at diagnosis and six months after completion of first-line treatments with CTC enrichment, done using density gradient centrifugation and CTC detection based on immunocytochemistry and in situ hybridization with probes targeting fusion genes (*MECOM* and *HHLA1*) previously described to be associated to a stem-cell like phenotype [77]. A quarter of the patients were CTC-positive at baseline and 7.7% were CTC-positive at the second time point, with the mean number of CTCs lower after treatment. CTCs at baseline were prognostic for patients with complete surgical resection (OS multivariate analysis, HR = 2.72; 95%CI [1.34–5.52]). Identification of residual CTCs was more frequent for patients with platinum-resistant disease. Combination of immunocytological staining with multiplex RT-PCR detected CTCs in 90% of 109 patients, 51 of whom had measurements at three time points [78]. *EpCAM* and *ERBB2* expression in CTCs were correlated to platinum resistance and survival, whereas CA125 level was not. In another series of 31 patients with OvC, serial blood sample collection allowed CTC identification for all patients by using an enrichment and detection method focused on Cell Adhesion Matrix-avidity [69]. CTC kinetics was correlated to CA125 changes under treatment and during follow-up (*r*^2^ = 0.47; *p* < 0.0001). CTC increase during follow-up was associated with an increased risk of progression, with an odds ratio of 121.3 (*p* < 0.00001) vs. 14.4 (*p* < 0.001) for CA125. CTCs were more sensitive than CA125 for detecting response or relapse. Moreover, CTC rate changes seemed to occur 0 to 3 months before clinical evidence of response to chemotherapy.

#### 3.2.2. Qualitative Analyses

Considering their high prognostic value and ability to detect drug resistance or increased risk of progression, quantitative assessment of CTCs may be routinely used in the following years for prognostic evaluation and monitoring, provided that standardized technology-dependent thresholds are established. In the same way, phenotypic, histologic, and genotypic studies may lead to new functional studies related to CTC biology and metastatic spreading [50,85]. We have already mentioned that patients with CTCs expressing *ERCC1*, a well-known marker of platinum-resistance, have a poorer prognosis [76]. CTC expression of the gene coding for Cyclophilin 3, a receptor of cyclosporine A, was also described to be correlated to platinum resistance [66]. The folate-receptor alpha (FRα) has been described to be overexpressed in many epithelial cancers including OvC, and FRα-targeting compounds have been developed. A multi-tumor study of 46 patients with cancer, of whom six had OvC, used the ApoStream^®^ system (Precision for Medicine™, Houston, TX, USA) for CTC enrichment followed by immunostainings including a FRα antibody and laser capture cytometry [86]. FRα-positive CTCs could be identified in patients with non-small cell lung cancer, breast cancer, and four of six patients with OvC.

In addition to phenotypic expression, the histologic presentation of CTCs is gaining some importance in the understanding of CTC biology. Indeed, detection of CTCs by EpCAM/DAPI-positive selection and CD45-negative staining identified CTC clusters for 50 (primary disease) to 67% (recurrent tumor) of the 54 patients explored, with a correlation to platinum-resistance [74]. CTC clusters have a 23- to 50-fold increased metastatic potential compared to the same number of isolated CTCs [87]. CTC clustering confers specific changes in DNA methylation that promote stemness, drug resistance, and metastasis [88]. Finally, the heterogeneity in composition of these clusters of cells has recently been the center of attention. Notably the association between neutrophils and CTCs was shown to drive cell cycle progression within the bloodstream and to the expand drug resistance abilities and metastatic potential of CTCs [89]. Ex vivo culture of CTCs was successful for two of 54 patients in this study [74]. Cultured CTCs were still EpCAM+/DAPI+/CD45−. Moreover, cultured CTCs had a more rapid proliferation and were more sensitive to chemotherapy than the historical SKOV3 and OVCAR3 cell lines. These preliminary results of CTCs culture may lead to the development of individual in vitro models for therapies assessment. EMT-like CTCs isolated from 91 patients using the AdnaTest Ovarian Cancer and EMT-markers (PI3Kα, Akt-2 and Twist) could be observed for 30% of patients and were mutually exclusive to EMT-negative CTCs for more than 80% of cases [90]. While epithelial CTCs decreased after chemotherapy, EMT-like CTCs not only increased in patients with EMT-like CTCs-positivity at baseline but also appeared in some initially negative cases, with higher rates in patients with residual tumors after debulking surgery. This suggests that EMT-like CTCs are enriched after chemotherapy, perhaps because of the stem-cells characteristics of EMT-driven cells [91]. The use of a size-based enrichment method (MetaCell^®^, Ostrava, Czech Republic) followed by CTC culturing, cytomorphological characterization, and the gene expression analysis (qPCR) of selected genes, showed that *EpCAM*, *WT1*, *MUC1*, *MUC16*, *KRT7*, *KRT18*, and *KRT19* mRNA are differentially expressed in CTCs when compared to whole blood samples [57,92]. Gene expression of all these markers after CTC-enrichment may improve CTC detection in another set of 118 patients with advanced stage disease, blood samples were collected before surgery, and CTCs were isolated using MetaCell^®^, and cultivated in vitro [93]. Seventy-seven patients (65.2%) were CTC-positive. CTC detection was correlated to FIGO stage, grade, ascites, and residual disease after surgery. CTCs and CA125 levels seemed to be independent prognostic factors. The study suggested that CTCs may be associated with hematogenous spreading, whereas CA125 is a surrogate marker of peritoneal dissemination. Because they are not related to each other [94], both markers may be complementary for OvC monitoring.

Workflows dedicated to single CTC genomic profiling have also been developed but remains highly challenging. Characterization of single CTCs requires technologies sensitive enough to isolate individual cells and capture CTC heterogeneity and clonality [95]. Single CTC analyses may reveal unknown biological mechanisms correlated to carcinogenesis, CTC spreading, and resistance to treatments, mainly by single cell culture, and DNA and RNA sequencing [96,97]. Concerning OvC, few data have been published to date. In a small set, 15 single CTCs from three OvC patients were isolated by using CellCelector™ (ALS GmbH, Jena, Germany) and analyzed with a combination of epithelial (*EpCAM*, *CK5/7*, *MUC1*), EMT (*N-cadherin*, *Vimentin*, *Snai1/2*, *CD117*, *CD146*, *CD49f*), and stem-cells (*CD44*, *ALDH1A1*, *Nanog*, *SOX2*, *Notch 1/4*, *Oct4*, *Lin28*) transcripts identified by multiplex RT-PCR after CTC enrichment (AdnaTest OvarianCancerSelect) and detection (AdnaTest OvarianCancerDetect) [98]. Most of the analyzed CTCs (13/15) were positive for EMT markers. Only four of the CTCs expressed stem cell markers. Both inter-patient and intra-patient CTC heterogeneity was observed. However, these data remain scarce and deeper single CTC analyses are warranted to explore CTC biology and clinical utility for OvC management.

### 3.3. Exosomes and Circulating Cell-Free Micro RNAs

Beside ctDNA and CTCs, other circulating biomarkers have been studied to improve our understanding of OvC. Data related to circulating extracellular vesicles (or exosomes) are still preliminary but may deliver several crucial data regarding disease evolution. They notably contain miRNA.

Extracellular vesicles are physiologically secreted by living cells and released in the extracellular space or in the blood stream. Living tumor cells also secrete exosomes. These cells have been described to be involved in tumor development and metastatic spreading [99]. Because they contain nucleic acids (DNA, RNA, microRNA), proteins, and other metabolites, they are a promising area of interest. Like CTCs, circulating exosomes can be isolated by ultracentrifugation, density-based separation, and magnetic beads coated with antibodies for surface antigens such as EpCAM [100,101,102,103].

However, other technologies based on laser light scattering or nano-plasmonic sensors have also been developed [104,105]. Due to their logistic complexity, these technologies are not easy to implement for clinical purposes.

Concerning OvC, the serum level of exosomes seems to be higher in patients with OvC than in healthy controls [106]. This increase may be explained by the association between exosome biogenesis and hypoxia, secretory pathways upregulation, and TP53 alterations were all enhanced in cancer cells [107,108]. Some exosomal markers have been studied. Tyrosine receptor kinase B, which has been associated to progression and prognosis, can thus be identified in serum exosomes, suggesting that it may be a biomarker for OvC diagnosis [109]. High levels of exosomal *MALAT1*, a long non-coding RNA associated to cancer metastasis, were observed in metastatic cases of OvC and were correlated to a poor outcome [110].

Most articles related to exosomes and OvC are actually focused on exosomal micro RNAs (miRNAs), which represent only a small fraction of circulating miRNAs [111]. Beside exosomal miRNAs, circulating cell-free miRNAs have also been explored in various settings such as diagnosis, prognosis assessment, resistance to systemic therapies, and monitoring [80,112,113].

MicroRNAs are small non-coding RNAs involved in several physiological and pathological processes [114,115]. Their localizations close to oncogenes or tumor suppressor genes as well as epigenetic alterations may explain their impact on cancer initiation and development. Tumor miRNAs have been widely explored and several signatures associated to survival and response to treatment have been published. A small part of miRNAs are released from cells and are quite stable in the blood stream [116,117]. Cell-free miRNAs can be detected using qRT-PCR, microarray platforms, or NGS, all technologies now routinely used in translational research and clinical labs. Concerning OvC, several miRNA signatures have been published [118]. Some of them are specific to plasma from patients with OvC compared to healthy subjects or patients with benign tumors [119,120,121,122,123,124,125,126,127]

A recently published meta-analysis showed that multiple miRNAs panels are promising for screening and diagnosis with a combined diagnosis odds ratio of 30.06 (95%CI [8.58–105.37) [128]. Circulating miRNA levels can also be correlated to prognosis and survival. Some studies showed that low levels of selected miRNAs are associated with a worse outcome [124,129,130], whereas high levels of others are associated with shorter survival [106,131,132,133,134,135,136]

However, as mentioned in a comprehensive review, publications concerning circulating miRNAs and OvC are controversial, and guidelines concerning pre-analytic procedures and methods for quantification and characterization of miRNAs are needed to standardize findings [118].

### 3.4. Non-Blood-Based Liquid Biopsies

Blood is not the only source of cell-free nucleic acids. Cell-free and exosomal miRNAs retrieved from urine, ascites, or uterine lavage can also be used to distinguish patients with OvC from healthy women [137,138,139,140]. Somatic *TP53* and *BRCA1/2* mutations can be detected by analyzing cfDNA isolated from peritoneal fluids [141,142]. Proteomic profiling of ascite-derived tumor cells of chemo-naïve and chemo-resistant OvC patients showed that proteins involved in metabolic pathways, DNA repair, and energy metabolism pathways are higher in tumor cells from chemo-resistant cases [143]. Pap smear specimens from patients with gynecological cancer have also been used for cfDNA analysis. Massive parallel sequencing of OvC from 22 patients allowed the identification of somatic mutations for 41% of analyzed cases [144]. Assessment of an optimized assay for 245 OvC patients showed a 33% sensitivity and 99% specificity when compared to healthy women [145]. Sensitivity was improved to 63% when the Pap test was combined to plasma ctDNA evaluation. Finally, sequencing of DNA retrieved from uterine lavage from 65 patients including 30 OvC and 27 benign gynecologic lesions showed that *TP53* mutation can be identified with 80% sensitivity [146]. No *TP53* mutation was observed in benign lesions, suggesting that uterine lavage may discriminate OvC from benign ovarian tumors. No data have been published concerning the value of *TP53* for OvC screening.

## 4. Conclusions

Publications related to liquid biopsies and OvC have exponentially increased since 2010. The ability of such biopsies to be repeated at several time points and recent technological advances allowing early detection of OvC-related abnormalities will likely lead to their use in clinical practice in the next few years, notably for treatment monitoring, detection of minimal residual disease, and early diagnosis of relapse using ctDNA and CTCs (Table 3). Extension to other settings, like screening and early diagnosis, will require further exploration. Nevertheless, their clinical validity and utility should first be proven, simplified and costs should be reduced before they may be widely recommended and routinely available. Results of prospective cohort studies and randomized trials using liquid biopsies for theragnostic purposes are thus warranted.

## Figures and Tables

**Table 1:** Published works concerning circulating tumor DNA and cell-free DNA analyses for patients with ovarian epithelial cancer. OvC, ovarian cancer; PCR, polymerase chain reaction; RT-PCR, reverse transcription polymerase chain reaction; NGS, next generation sequencing; PFS, progression-free survival; OS, overall survival.

Reference	Areas of Interest	ctDNA /cfDNA	Methods	Molecular Alterations	*N*	FIGO Stage	Results
[28]	Screening /Diagnosis	cfDNA	Low coverage whole genome sequencing	Chromosomal instability	68 adnexal masses 44 benign cases	I–II = 8 III–IV = 46 Borderline = 3 Benign = 11	Chromosomal instability significantly more elevated in tumor cases than healthy and benign cases
[22]	Screening /Diagnosis	cfDNA	Low coverage whole genome sequencing	Copy number alterations	32 OvC 32 benign cases		Detection rate 40% (13/32) Sensitivity 40.6% Specificity 93.8%
[29]	Screening /Diagnosis Prognosis	ctDNA	Droplet digital PCR	Tumor specific mutations	44 gynecological cancers including 22 OvC		Detection rate 93.8% Sensitivity 91%, Specificity 60% Undetectable ctDNA 6 months after treatment completion was associated to a better PFS (*p* = 0.0011) and OS (*p* = 0.0194).
[30]	Screening /Diagnosis	cfDNA	DNA microarray	Methylation profiling *PGR-PROX* and *RASSF1A*	30 OvC 30 benign cases	III–IV	Sensitivity 80% and Specificity 73%
[31]	Prognosis	cfDNA	Fluorescence	Fluorimetry	62	I–II = 18% III = 69% IV = 13%	Pre-operative ctDNA is correlated to PFS (*p* = 0.02) and OS (*p* = 0.01)
[32]	Prognosis	cfDNA	RT-PCR	Beta-globin	164	I–II = 38 III–IV = 126	Pre-treatment ctDNA levels are correlated to OS (median 3.1 years for high ctDNA levels vs. 4 years for patients with low ctDNA rate.
[33]	Prognosis	ctDNA	PCR	Methylation profiling	138		Correlation between *hMLH1* methylation acquisition after chemotherapy and post-progression survival (HR = 99; *p* = 0.007)
[34]	Prediction and monitoring of response to treatment	ctDNA	Digital PCR	*TP53* mutations	40 (mostly relapses)	I = 3 III = 27 IV = 10	*TP53* mutant allele fraction associated to tumor burden and time to progression (HR = 0.22, *p* = 0.008, multivariate analysis). ctDNA is an earlier marker of response to treatment (median time to nadir of 37 days vs. 84 days for CA125)
[35]	Prediction and monitoring of response to treatment	ctDNA	Droplet digital PCR	*TP53* mutations	41	III–IV	Correlation between plasma *TP53* mutant allele count 3 months after chemotherapy completion and time to progression (*p* = 0.038)
[36]	Prediction and monitoring of response to treatment	ctDNA	NGS	*BRCA1/2* mutations	121		Mutations detected for 24.8%
[37]	Prediction and monitoring of response to treatment	ctDNA	NGS, Targeted amplicon deep sequencing	*TP53* mutant allele fraction	18	Relapses	0/5 patients low ctDNA decrease achieved radiological response. 7/9 patients with high ctDNA decrease were responder
[38]	Prediction and monitoring of response	ctDNA	NGS, Targeted amplicon-sequencing	*BRCA1/2* and *TP53*	30	II = 2 III = 23 IV = 5	*BRCA1/2* reversion mutation identified in 5 tumors and 3 plasma samples
[39]	Prediction and monitoring of response	ctDNA	NGS	Panels of genes (Guardant360 and FundationACT)	112		*BRCA1/2* mutation detected in 97 patients. *BRCA1/2* reversion mutation found in pre-treatment ctDNA from 8 patients. Correlation to PFS after rucaparib (median 9 months vs. 1.8 months (*p* < 0.0001).
[40]	Prediction of response	ctDNA	NGS	143-gene panel	19	Platinum-resistant	*BRCA1/2* reversion mutations identified for 4 (21%) patients with OvC
[41]	Prediction and monitoring of response	ctDNA	qRT-PCR	*FGFR2* transcript fusion	1	IIIc	Systematic increase of transcript at each relapse with better sensitivity than CA125
[42]	Monitoring of response	ctDNA	qPCR	Tumor specific chromosomal rearrangements	10	IIIc and IV	8 cases with rearrangements identified in plasma at diagnosis. Correlation between persistence of detectable rearrangements and post-operative residual disease.

**Table 2:** Main published works concerning circulating tumor cells analyses for patients with ovarian epithelial cancer.

Reference	Areas of Interest	CTC Enrichment Methods	CTC Detection Methods	*N*	FIGO Stage	Results
[66]	Diagnosis	Density gradient centrifugation	RT-qPCR	39 healthy subjects$216 OvC	II = 8 III = 152 IV = 40	24.5% of patients with OvC were CTC+ vs. 1/39 of healthy subjects
[67]	Diagnosis Prognosis	Cell adhesion matrix-based	Microscopy and flow cytometry (EPCAM, CA125, DPP4, CD44, seprase, CD45, cytokeratins)	41 benign masses 88 OvC	I = 13 II = 4 III = 50 IV = 21	83% sensitivity 97.3% positive predictive value Correlation with OS and PFS higher than CA125
[68]	Screening /Diagnosis	Physical properties (TSF platform)	Immunostaining (DAPI+, CD45−, CK+ or EpCAM+)	43 benign cases 13 border line tumors 31 OvC	I = 6 II = 4 III-IV = 21	77.4% sensitivity and 100% specificity to discriminate benign from malignant tumors
[69]	Prediction of response Early diagnosis of relapse	Cell adhesion matrix-based	Microscopy and flow cytometry (EPCAM, CA125, DPP4, CD44, seprase, CD45, cytokeratins)	64 healthy subjects 49 benign cases 123 OvC (including 31 with serial time points)	I = 9 II = 1 III = 13 IV = 8	Higher correlation to duration of response than CA125 CTCs more sensitive than CA125 to predict relapse
[70]	Staging Prognosis	Density gradient centrifugation	Immunostaining (CD45−, CK+, ESA+ or EpCAM+)	66 OvC 5 benign masses	I–II = 10 II = 37 IV = 15	Sensitivity from 10% (stage I–II) to 73% (III–IV) Correlation with DFS (median 15 months for CTC+ vs. 35 months for CTC−, *p* = 0.042
[71]	Prognosis	Immunomagnetic assay (EpCAM/MUC1)	RT-qPCR (*EpCAM/MUC1/ERBB2*)	122 OvC	86 pre-chemo 70 post-chemo	CTC+ correlated with OS, both for pre-chemo samples (*p* = 0.0054) and after chemotherapy (*p* = 0.047).
[72]	Prognosis	Immunomagnetic assay (EpCAM/MUC1)	RT-PCR *(EpCAM/MUC1/* *MUC16/ERCC1)*	143	I–II = 26 II = 87 IV = 30	CTC+ correlated to OS. CTC ERCC1+ correlated to OS, PFS, and platinum resistance.
[73]	Prognosis	CellSearch	Immunostaining (DAPI+, EpCAM+, CD45−)	216	Relapses (66.2% platinum-sensitive)	Trend for CTC correlation to PFS (*p* = 0.058) and OS (*p* = 0.096)
[74]	Prognosis	Polymer-deposited microfluidic device	Immunostaining (DAPI+, EpCAM+, CD45−)	54	24 primaries 30 relapses	≥3 CTCs correlated to PFS CTC-cluster+ correlated with platinum resistance
[75]	Prognosis	CellSearch	Cellsearch (DAPI+, EpCAM+, cytokeratin+, CD45−)	54	All relapses	CTC+ correlated to lack of response to temsirolimus
[76]	Minimal residual disease	AdnaTest Ovarian Cancer Select	RT-PCR (AdnaTest Ovarian Cancer Detect, *ERCC1*)	65	I–II = 11 III = 41 IV = 13	ERCC1 + CTCs after chemotherapy correlated with platinum-resistance (*p* = 0.01), PFS (*p* = 0.0293) and OS (*p* = 0.0008)
[77]	Prognosis Prediction of response	Density gradient centrifugation+	IHC+ fusion genes associated to stem-cell like phenotype *(MECOM, HHLA1)*	Blood collected at diagnosis (*n* = 102) + 6 months after treatment completion (*n*= 78)	II = 4 III = 67 IV = 31	26.5% CTC+ at baseline 7.7% CTC+ after treatment CTC status at baseline associated to survival for patient with complete debulking surgery CTC status after treatment correlated to response to platinum
[78]	Prediction of platinum resistance Prognosis	Immunocytological staining	Multiplex RT-PCR	109 at diagnosis, including 51 at serial time points	I = 23 II = 13 III = 58 IV = 15	90% CTC+ at baseline Higher sensitivity than CA125 for early stages *EpCAM* and *ERBB2* correlated to platinum resistance *EpCAM* correlated to survival

**Table 3:** Comparison of circulating biomarkers for ovarian cancer analysis.

Heading	Circulating Tumor DNA	Circulating Tumor Cells	Extracellular Vesicles (Exosomes)	Tumor Micro-RNA
**Origins**	DNA released by apoptotic or necrotic cells, carrying specific molecular alterations of the tumor. Complementary to tumor genomic profiling (due to spatial heterogeneity and possibility of serial blood sampling).	Cells released in the bloodstream by the primary tumor or metastatic sites. Some of them are able to colonize distant sites after epithelial to mesenchymal transition.	Physiologically secreted by cells and released in the extracellular space and the bloodstream. Contain nucleic acids (DNA, RNA, micro-RNA), proteins, and metabolites	Carried by exosomes or isolated in the plasma (cf-miRNA). Highly stable in serum/plasma
**Settings of interest**	Screening/early diagnosis Prognosis Identification of minimal residual disease and monitoring of response to treatment	Screening/early diagnosis Prognosis Response to treatment Evaluation of minimal residual disease Functional studies (phenotypic and genotypic studies)	Diagnosis Prognosis	Screening/diagnosis Prognosis
**Methods**	**Blood sample collection and processing** • EDTA tubes - Widely available, low cost - Risk of degradation of DNA and release of genomic DNA from hemotopoietic cells. Warrant rapid processing (1 to 2 h from collection) • cfDNA preservative tubes: - More expensive - Maintain the quality of DNA for multiple days at room temperature. **DNA extraction from plasma** (kits designed for fragmented DNA) **Analysis** • **Targeted methods:** Droplet digital PCR, BEAMing (beads, emulsion, amplification, magnetics) - Focus on individual predefined alterations. Highest sensitivity • **Untargeted methods:** NGS - Sequencing of large panel of genes. Ability to detect several alterations simultaneously. - High sensitivity with adapted technologies (barcoding) and specific computational pipelines (ex: TecSeq (Targeted error correction Sequencing), TamSeq (Tagged amplicon Sequencing), or CAPP-Seq (CAncer Personalized Profiling by deep Sequencing).	**Blood sample collection** CTC’s half-life is shorter than ctDNA after blood collection (around 4 h). Blood samples have to be processed quickly. **Enrichment** • Biophysical features: size, density, electric charges, deformability, invasive capacity • Biological features: expression of epithelial markers and absence of expression of hematopoietic markers (CD45 for example) **Detection** • Immune-cytological assays (epithelial protein expression) • Genomic assays (epithelial mRNA) • Functional methods **Limits**: Warrant specific platforms, limiting its use in routine practice.	**Isolation** • Ultracentrifugation • Density-based separation • Magnetic beads coated with antibodies for surface antigens • Laser-light scattering • Nano-plasmonic sensors **Issue**: High logistic complexity limiting routine clinical use	**Isolation** • Commercially available kits based on TRIzol or column-based extraction **Detection** • qRT-PCR • microarrays • NGS **Issue**: lack of standardized pre-analytic and analytic procedures

## Supplementary Materials

The following are available online at https://www.mdpi.com/2072-6694/11/6/774/s1, Figure S1: PRISMA diagram, Table S1: PRISMA checklist.
